# A strategy for high antibody expression with low anti-drug antibodies using AAV9 vectors

**DOI:** 10.3389/fimmu.2023.1105617

**Published:** 2023-04-21

**Authors:** Meredith E. Davis-Gardner, Jesse A. Weber, Jun Xie, Katja Pekrun, Eric A. Alexander, Kim L. Weisgrau, Jessica R. Furlott, Eva G. Rakasz, Mark A. Kay, Guangping Gao, Michael Farzan, Matthew R. Gardner

**Affiliations:** ^1^ Center for Childhood Infections and Vaccines of Children’s Healthcare of Atlanta, Department of Pediatrics, Emory University, Atlanta, GA, United States; ^2^ Department of Immunology and Microbiology, University of Florida (UF) Scripps Biomedical Research, University of Florida, Jupiter, FL, United States; ^3^ Horae Gene Therapy Center, University of Massachusetts Medical School, Worcester, MA, United States; ^4^ Department of Microbiology and Physiological Systems, University of Massachusetts Chan Medical School, Worcester, MA, United States; ^5^ Departments of Pediatrics and Genetics, Stanford University, Stanford, CA, United States; ^6^ Wisconsin National Primate Research Center, University of Madison-Wisconsin, Madison, WI, United States; ^7^ Department of Medicine, Division of Infectious Diseases, Emory University, Atlanta, GA, United States; ^8^ Division of Microbiology and Immunology, Emory National Primate Research Center, Emory University, Atlanta, GA, United States

**Keywords:** AAV (adeno-associated virus), HIV - human immunodeficiency virus, SIV - simian immunodeficiency virus, antibody, anti-drug antibodies (ADA)

## Abstract

**Introduction:**

Use of adeno-associated virus (AAV) vectors is complicated by host immune responses that can limit transgene expression. Recent clinical trials using AAV vectors to deliver HIV broadly neutralizing antibodies (bNAbs) by intramuscular administration resulted in poor expression with anti-drug antibodies (ADA) responses against the bNAb.

**Methods:**

Here we compared the expression of, and ADA responses against, an anti-SIV antibody ITS01 when delivered by five different AAV capsids. We first evaluated ITS01 expression from AAV vectors three different 2A peptides. Rhesus macaques were selected for the study based on preexisiting neutralizing antibodies by evaluating serum samples in a neutralization assay against the five capsids used in the study. Macaques were intramuscularly administered AAV vectors at a 2.5x10^12 vg/kg over eight administration sites. ITS01 concentrations and anti-drug antibodies (ADA) were measured by ELISA and a neutralization assay was conducted to confirm *ex vivo* antibody potency.

**Results:**

We observed that ITS01 expressed three-fold more efficiently in mice from AAV vectors in which heavy and light-chain genes were separated by a P2A ribosomal skipping peptide, compared with those bearing F2A or T2A peptides. We then measured the preexisting neutralizing antibody responses against three traditional AAV capsids in 360 rhesus macaques and observed that 8%, 16%, and 42% were seronegative for AAV1, AAV8, and AAV9, respectively. Finally, we compared ITS01 expression in seronegative macaques intramuscularly transduced with AAV1, AAV8, or AAV9, or with the synthetic capsids AAV-NP22 or AAV-KP1. We observed at 30 weeks after administration that AAV9- and AAV1-delivered vectors expressed the highest concentrations of ITS01 (224 µg/mL, n=5, and 216 µg/mL, n=3, respectively). The remaining groups expressed an average of 35-73 µg/mL. Notably, ADA responses against ITS01 were observed in six of the 19 animals. Lastly, we demonstrated that the expressed ITS01 retained its neutralizing activity with nearly the same potency of purified recombinant protein.

**Discussion:**

Overall, these data suggest that the AAV9 capsid is a suitable choice for intramuscular expression of antibodies in nonhuman primates.

## Introduction

With the FDA approval of Luxturna in 2017 and Zolgensma in 2019, adeno-associated virus (AAV) vectors are poised to lead to new breakthrough gene therapies in the coming years. There are numerous applications of AAV gene therapy for treating genetic and infectious diseases; and thus, selection of the best AAV capsid is crucial for achieving positive therapeutic outcomes. Selection of an ideal AAV capsid also relies on route of administration of the specific tissue target to achieve efficient transduction that yields therapeutic expression of the transgene.

While efforts to develop a traditional HIV-1 vaccine are ongoing, numerous broadly neutralizing antibodies (bNAbs) have been identified and characterized (reviewed in (1) and (2)). Due to the inherent difficulties in eliciting HIV-specific bNAbs by conventional vaccination strategies, AAV-delivered bNAbs or HIV-1 inhibitors provide a promising alternative strategy to achieve durable anti-HIV immunity after a single vector adminstration (3–9). We and others have been deploying this approach for bNAbs and HIV-1 inhibitors through intramuscular (i.m.) administration with AAV vectors. Skeletal muscle cells are an ideal cell type for this strategy because of their long lifespan, which enables the antibody or inhibitor to be expressed for years (10), possibly decades.

Our studies, along with others, using AAV vectors for i.m. administration have primarily used the AAV1 capsid as it has been described to have muscle tissue tropism (4, 5, 8, 11–14). Others have successfully used the AAV8 capsid for i.m. administration (3, 6, 7, 15, 16). One study has shown AAV8 to be less immunogenic than AAV1 and therefore could be a more promising capsid for these purposes (17). Additionally, the AAV9 capsid has been shown to successfully transduce muscle tissue and express antibodies and inhibitors at very high concentrations in macaques (18). Engineered capsids have also been developed for muscle tissue transduction or other cell types that have also shown promise for muscle cell transduction. Two of these include AAV-NP22 and AAV-KP1 (19, 20). Despite there being numerous options for capsid selection for i.m. administration, no study has ever compared multiple capsids in non-human primates before. Furthermore, two recent clinical studies using AAV1 to deliver PG9 (11) and AAV8 to deliver VRC07 (16) resulted in no or low detectable concentrations of the delivered bNAbs.

In this study we test the three traditional AAV capsids – AAV1, AAV8, and AAV9 – along with two engineered capsids – AAV-NP22 and AAV-KP1 – for expression of the anti-SIV antibody, ITS01 (15), after i.m. administration. We first optimized expression of ITS01 from an AAV transgene cassette by identifying that the P2A peptide leads to significantly higher ITS01 expression in mice when compared to the F2A and T2A peptides. When assessed in macaques, the AAV9-inoculated animals expressed the highest serum concentrations over a 30-week period. While only six of the 19 macaques developed ADAs against expressed ITS01, in only one macaque did this coincide with a decrease in ITS01 concentrations. Taken together, AAV9 is a suitable capsid for promoting persistent antibody expression from muscle cells, especially when the antibody is “self-like” to not stimulate an immune response.

## Results

### Optimization and characterization of AAV-expressed ITS01

**Figure 1:**
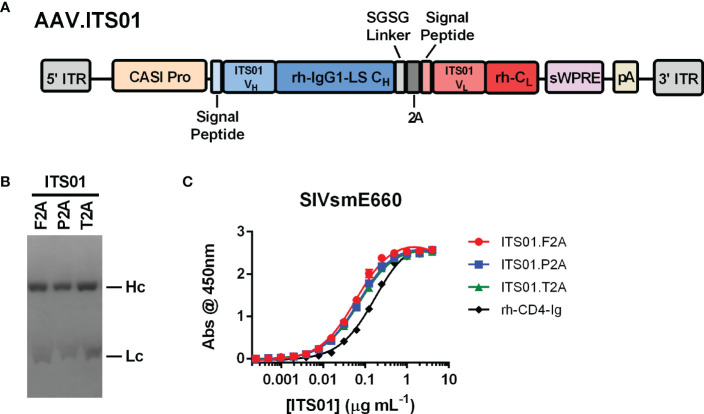
Schematic and characterization of ITS01 expressed from an AAV transfer plasmid. **(A)** Schematic of an the AAV vector encoding ITS01 from 5’ ITR to 3’ ITR. ITS01 expression is driven by a CASI promoter and utilizes a 2A peptide to generate both the heavy and light chains. **(B)** Coomassie-stained SDS-PAGE of purified ITS01 antibodies generated with the indicated 2A peptide. **(C)** SIVsmE660-coated ELISA plates were incubated with indicated concentrations of purified recombinant ITS01 antibody. ITS01 binding was determined using an HRP-conjugated, anti-human Fc secondary antibody and binding activity was measured at an absorbance of 450 nm. ITS01 antibody made using F2A, P2A, and T2A peptide is indicated. rh-CD4-Ig is a positive control protein. Error bars indicate standard deviation (S.D.).

To test expression of an antibody from an AAV vector, we selected the anti-SIV antibody ITS01, a 2A peptide design, and CASI promoter (**Figure 1A**). ITS01 is about 5-6% divergent from its closest germline precursor antibody in the rhesus macaque antibody repertoire (15) and binds to the SIV envelope glycoprotein the SIVsmE660 isolate. When expressed from an AAV vector in rhesus macaques, ITS01 has shown to have lower ADA responses compared to studies using HIV-1 bNAbs (15). The CASI promoter has demonstrated to be a robust promoter for expressing antibodies in mice *via* i.m. administration (7). The 2A design has also been a widely used strategy for expressing a full-length antibody from a single expression cassette (21–23). Previous designs utilized the F2A peptide but other studies have shown that the P2A and T2A peptides may be better (21, 23). Thus, we first set out to down-select on a 2A peptide. AAV transfer plasmids were engineered to express the ITS01 heavy and light chains using the F2A, P2A, or T2A peptides. Purified ITS01 protein harvested from transiently transfected HEK293T cells had similar cleavage efficiencies when assessed by Coomassie-stained SDS-PAGE (**Figure 1B**) and binding efficiencies on SIVsmE660 gp120 protein as measured by ELISA (**Figure 1C**).

**Figure 2:**
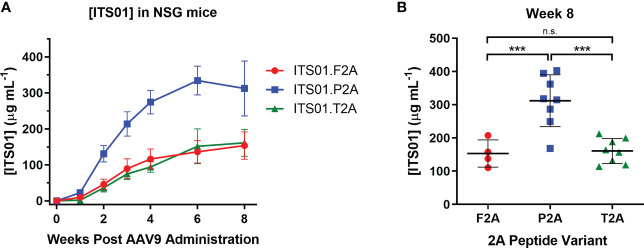
ITS01 expression from AAV9 vectors using different 2A peptides in NSG mice. **(A)** Three groups of four (F2A) or eight (P2A and T2A) NSG mice each were administered a fixed dose of 5×10^10^ vector genomes (vg) AAV9 vectors (about 2.5×10^12^ vg/kg) encoding ITS01 using the indicated 2A peptide (F2A – red, P2A – blue, T2A – green) in a 25 µL volume in the left gastrocnemius muscle. Blood draws were performed over eight weeks and plasma samples were analyzed by gp120 ELISA to determine ITS01 concentrations. **(B)** Comparison of ITS01 concentrations at week 8 post AAV administration. Each dot represents an individual animal. Mean is represented by the black bar. Error bars indicate S.D. Statistical comparison of the 2A peptide groups was determined by a one-way ANOVA with Tukey’s multiple comparison test. Statistical significance is defined as *** indicates P value ≤ 0.001; n.s., indicates not significant.

To down-select on a 2A peptide for generating ITS01, we tested expression of ITS01 from intramuscular administration of NSG (NOD.Cg-*Prkdc^scid^* Il2rg^tm1wjl^/SzJ or NOD-*scid* IL2Rgamma^null^) mice. Purified rAAV9 vectors encoding ITS01 using one of the three 2A peptides were intramuscular inoculated into the left gastrocnemius muscle at a fixed dose of 5×10^10^ vector genomes (vg) or about 2.5×10^12^ vg/kg. Plasma concentrations of ITS01 were tracked for eight weeks post administration by gp120 ELISA (**Figure 2A**). We observed robust expression in all three groups as all 20 mice had >100 µg/mL of ITS01 in the plasma by the end of the study. However, at eight weeks post administration, we observed an average two-fold increase in ITS01 concentration in the P2A group (range 168 – 402 µg/mL) compared to both the F2A and T2A groups (**Figure 2B**). Based on this study, we selected the P2A version of the expression cassette to use in the nonhuman primate (NHP) study.

### Selection of rhesus macaques based on preexisting AAV neutralizing antibodies

**Figure 3:**
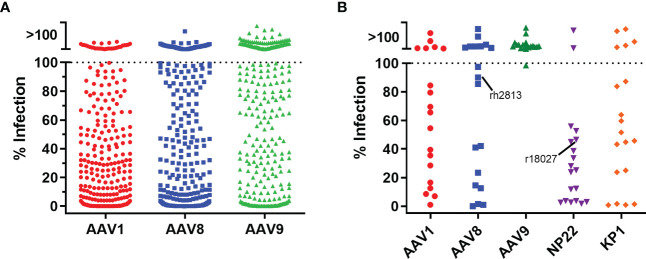
Pre-screening rhesus macaques for AAV neutralizing antibodies. **(A)** To screen for neutralizing antibodies, we utilized a cellular assay similar the HIV-1 TZM-bl neutralization assay. 360 rhesus macaque serum samples were diluted 1:5 in cell culture medium and mixed at a 1:1 ratio (final serum dilution of 1:10) with AAV vectors encoding the firefly luciferase reporter. After 60 minutes, 3×10^4^ cells were added to the serum/virus mixture and incubated for 24 hours. Neutralization was determined as the absence of luciferase production. We observed that 8%, 16%, and 42% of the samples were negative for neutralizing antibodies against AAV1, AAV8, and AAV9 capsids, respectively. **(B)** 19 rhesus macaque samples for the NHP study were screened for neutralizing responses as in **(A)** except that AAV-NP22 and AAV-KP1 vectors were included. Three macaques that were negative for each capsid were selected to be used in the study, except for AAV-NP22, which only had two macaques without pre-existing neutralizing antibodies. The third macaque selected, r18027, is identified with about a 50% neutralizing response to AAV-NP22. The fifth macaque selected for the AAV8 group, rh2813, had 10% neutralizing activity at a 1:10 dilution.

With a transgene cassette design selected, we next evaluated five different AAV capsids for ITS01 expression from i.m. administration in rhesus macaques. While our previous studies utilized the AAV1 capsid for i.m. administration, other studies suggested that AAV8 or AAV9 vectors could be suitable for i.m. administration (3, 6, 7, 15, 16). For all our previous studies in rhesus macaques, we selected macaques that were absent of preexisting neutralizing antibodies to avoid any possible interference with AAV vector transduction. Interestingly, when we assayed 360 rhesus macaque samples from five Primate Research Centers in the United States for preexisting anti-AAV neutralizing antibodies, we observed no detectable neutralizing activity at a 1:10 serum dilution against AAV1, AAV8, and AAV9 in 8%, 16% and 42% of the macaque samples, respectively (**Figure 3A**). Additionally, two recent synthetic capsids generated though capsid shuffling, AAV-NP22 and AAV-KP1, were also described to be potentially better capsids than AAV1 for i.m. administration (19, 20). For our study, we screened 19 macaques for preexisting neutralizing responses against AAV1, AAV8, AAV9, AAV-NP22, and AAV-KP1. We were able to identify at least three macaques to be grouped for each capsid that had no neutralizing activity, except for AAV-NP22 (**Figure 3B**). For the AAV-NP22 group, we included r18027, which had preexisting neutralization activity at 54% at a 1:10 serum dilution. Additionally, when increasing the AAV8 group size, we included rh2813, which had 10% neutralizing activity at a 1:10 serum dilution.

### Evaluation of AAV-expressed ITS01 in rhesus macaques with five AAV capsids

**Figure 4:**
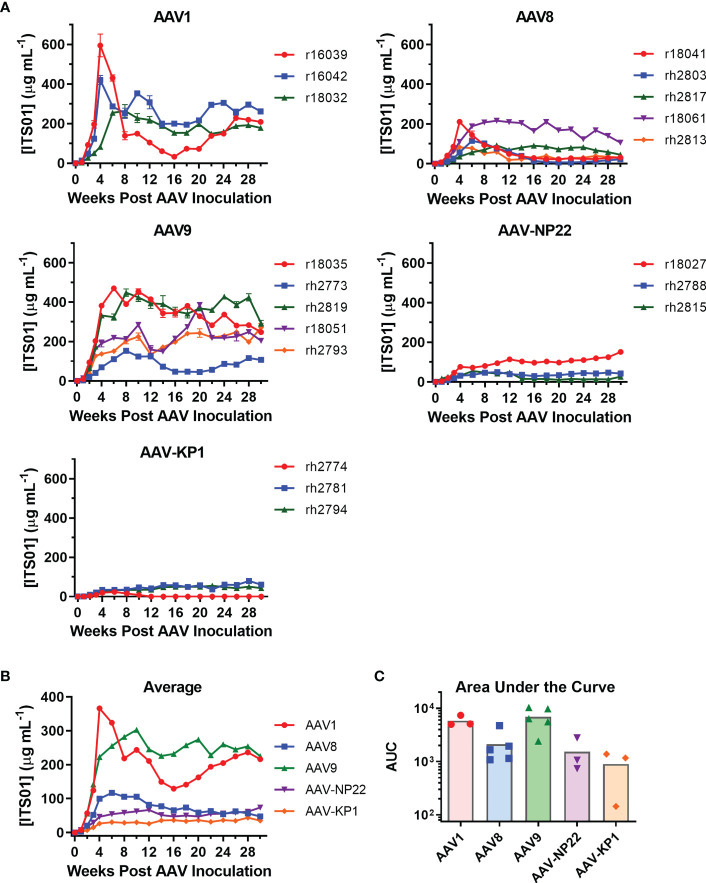
Comparison of ITS01 concentrations from rhesus macaques with different AAV vectors. **(A)** Quantification of ITS01 concentration from AAV vectors with AAV1, AAV8, AAV9, AAV-NP22, and AAV-KP1 capsids. Five groups of three or five macaques each were inoculated with 2.5×10^12^ vg/kg of the indicated AAV vector in eight injection sites per macaque. ITS01 concentrations for the AAV1, AAV8, AAV9, AAV-NP-22, and AAV-KP1 groups were measured over the course of 30 weeks by SIVsmE660 gp120 ELISA. **(B)** The average ITS01 concentrations for each group are represented. **(C)** The average area under the curve (AUC) as for each capsid group is represented with each individual icon representing an animal from that capsid group. Error bars indicate S.D. in **(A, B)**.

The main goal of our study was to select a capsid for i.m. administration. With our five capsids selected, three groups included three macaques each (AAV1, AAV-NP22, and AAV-KP1) and two groups had five macaques each (AAV8 and AAV9). The AAV8 and AAV9 groups were larger for better comparisons as they are two capsids of current clinical interest. AAV vectors were quality controlled through negative-stain transmission electron microscopy for full/empty particle analysis (**Figures S1A, B**). Each vector prep resulted in 85% to 95% full vector particles. All 19 macaques received i.m. administration of their respective AAV vector encoding ITS01 at a dose of 2.5×10^12^ vg/kg. The injections were spread out evenly over eight injection sites: two per quadriceps muscle, one in each bicep muscle, and one in each deltoid muscle. We quantified ITS01 expression from serum samples taken over the course of 30 weeks by gp120 ELISA. ITS01 concentrations for the individual macaques in each group are shown in **Figure 4A** and the average ITS01 concentrations for each group are shown in **Figure 4B**. When specifically looking at the two animals that were measured to have some preexisting neutralizing activity against the AAV vector administered, rh2813 and r18027 resulted in 31 and 151 µg/mL at week 30. This would suggest that the neutralizing activity present did not impact AAV vector transduction as both animals had sustained ITS01 concentrations throughout the study. Finally, as also shown by area under the curve (AUC) analysis (**Figure 4C**), the AAV9 group had the highest average ITS01 concentrations throughout the study with an average concentration ranging from 224 to 302 µg/mL starting at week 10 post administration. Despite having the highest peak, the AAV1 group had the second-best average concentration and AUC, with the average ranging from 216 to 243 µg/mL after week 10. The AAV9 and AAV1 groups were noticeably better than the AAV8, AAV-NP22, and AAV-KP1 groups, the latter three having average ITS01 concentrations less than 100 µg/mL. We also noted that there was no correlation for ITS01 concentration (AUC) and injection volume (**Figure S2**, **Table S1**) These results demonstrate that vectors utilizing the AAV9 capsid are just as good, if not better, than AAV1 encapsidated vectors at transducing skeletal muscle tissue in rhesus macaques.

**Figure 5:**
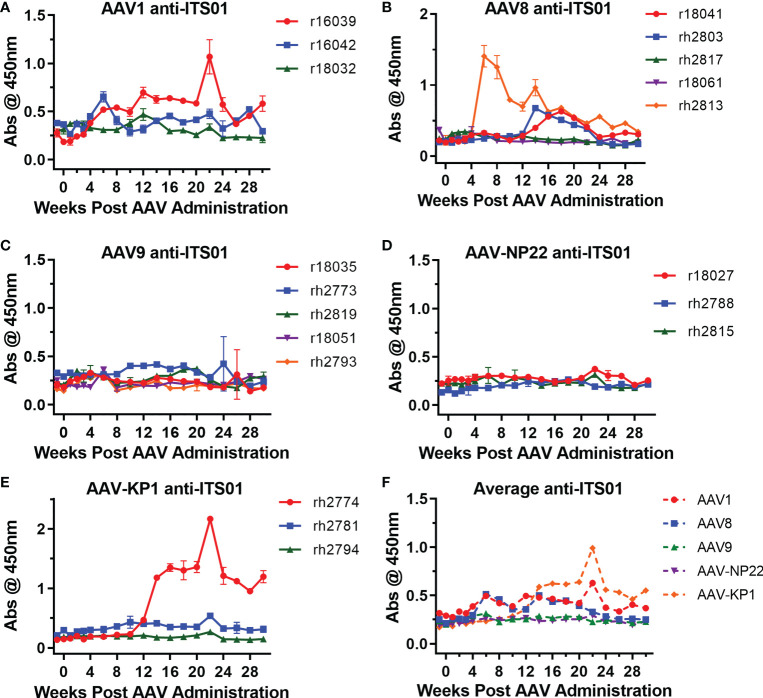
Low ADA responses against expressed ITS01 in all groups. ADA responses were measured by ELISA. ITS01-coated plates were incubated with diluted serum samples from the incubated timepoints. Anti-ITS01 antibodies were detected using a human, anti-lambda secondary. **(A–E)** The absorbance values for each macaque from the study are graphed based on capsid group. Error bars indicate S.D. **(F)** Average absorbance values for the five groups. Overall, 13 of 19 macaques have ADA responses around the absorbance values of the pre-administration time points.

One issue that continues to plague the AAV-expressed HIV-1 antibody work in rhesus macaques is the development of ADA responses, which usually correlate with lower expression of the HIV-1 antibody (3, 5, 11, 12, 14). Therefore, we next analyzed whether ADAs were generated against ITS01, an anti-SIV antibody derived from a rhesus macaque. Encouragingly, 13 of the 19 macaques in this study did not develop ADAs detectable above background absorbance values taken before the AAV administration (**Figures 5A–E**), despite samples being tested at a 1:10 dilution. Overall, two macaques in the AAV1 group (r16039 and r16042), three in the AAV8 group (r18041, rh2803, and rh2813), none in the AAV9 and AAV-NP22 groups, and one in the AAV-KP22 group (rh2774) had ADAs above background at least one time point during the study, a total six macaques out of 19 total (31.6%). **Figure S3** plots the ITS01 concentrations vs. ADA for each individual macaque. In only one instance, did the appearance of ADAs coincide with a decrease in ITS01 concentrations to <1 µg/mL, which was rh2774 in the AAV-KP1 group. Out of the other five macaques that had ADA against ITS01, r16039, r18041, rh2803, rh2813 appeared to have ITS01 concentrations that decreased over time. However, for these macaques, ITS01 remained measurable throughout the study, all finishing with an ITS01 concentration >20 µg/mL. When comparing the average absorbance values for each group throughout the study, we did not observe a noticeable difference between the groups (**Figure 5F**). Based on these data, we conclude that when an AAV-delivered antibody is minimally mutated compared to its germline precursor, ADA may not be a concern.

### AAV-expressed ITS01 retains potency in ex vivo neutralization assays

**Figure 6:**
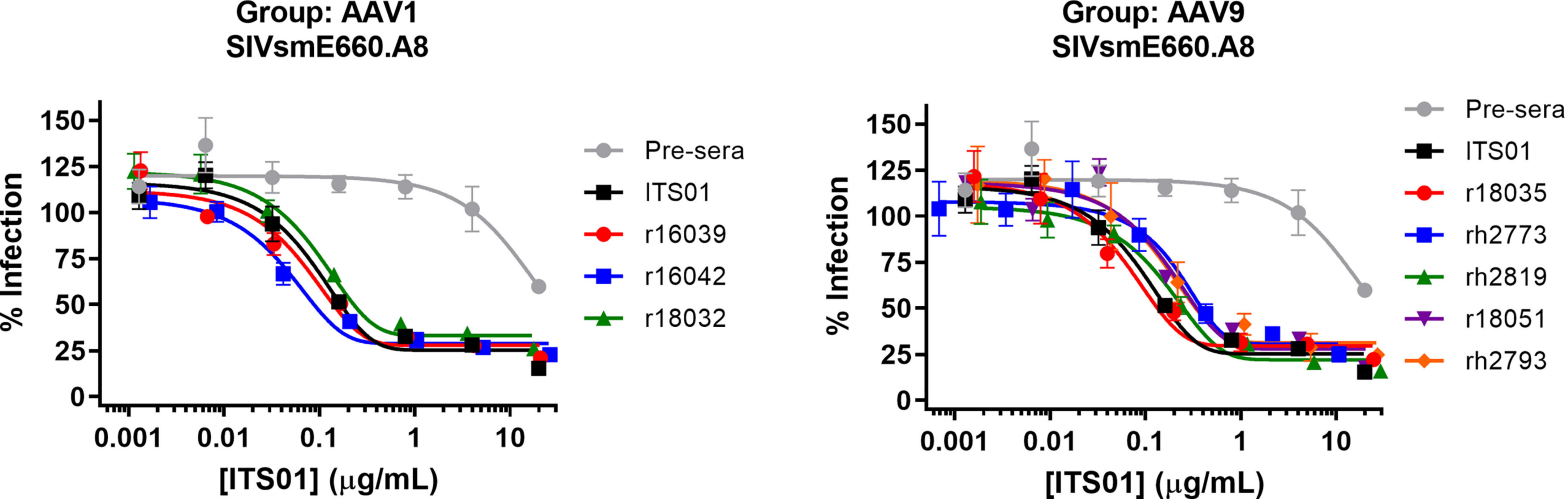
ITS01 from macaque serum retains its neutralizing activity in *ex vivo* neutralization assays. Neutralization activity of serum samples from 30 weeks post AAV administration for the AAV1 (left) and AAV9 (right) macaques against SIVsmE660.A8 pseudovirus was assessed by TZM-bl neutralization activity. A serum sample from before AAV vector administration was used as a negative control and the same serum sample with a known concentration of ITS01 was used as a positive control. Error bars indicate S.D.

Lastly, we analyzed the serum samples to ensure they maintained neutralizing activity. Sera from the week 30 timepoint of the AAV1 and AAV9 groups were initially diluted 1:10 in media then serially diluted 5-fold and mixed with HIV-1 pseudotyped with the SIVsmE660.A8 envelope glycoprotein (**Figure 6**). TZM-bl cells were used to quantify neutralization activity. We observed that all eight samples from both the AAV1 and AAV9 groups had potency against SIVsmE660.A8 that was nearly identical to naïve macaque serum spiked with a known concentration of purified, recombinant ITS01 antibody. These data demonstrate that the gp120 ELISA accurately determined the serum concentrations of ITS01 in rhesus macaques and that the expressed ITS01 antibody maintains its neutralizing function in blood.

## Discussion

In this study, we compared five AAV capsids for their ability to express an antibody after i.m. administration in rhesus macaques. While AAV1 has historically been the capsid of choice for i.m. administration, our data show that AAV9 can function as a suitable replacement. Surprisingly, both AAV1 and AAV9 capsids outperformed the AAV8 capsid for ITS01 expression by an average increase of about 4.6-fold 30 weeks post AAV administration. Additionally, the capsid selected for muscle transduction, AAV-NP22, and a second engineered capsid, AAV-KP1, performed in range of the AAV8 capsid group. We were able to rule out a few possibilities for differences such as full-empty capsid ratio (85% to 98% for each vector shown in **Figure S1B**), injection volume (no correlation shown in **Figure S2**), and day-to-day variation for quantifying vectors (all except the second batches of AAV-NP22 and AAV-KP1 were quantified on the same day). This may indicate that the engineered capsids might be better suited for transducing muscle cells after systemic administration rather than i.m. administration or there could be cellular factors on NHP muscle cells that are not present on the cell types used to select the engineered capsids.

These data also bring to our attention some limitations of this study. One limitation was that we did not obtain biodistribution data for the cells that were transduced by AAV vectors and expressing ITS01. Assessing the muscle tissues that received AAV injections would be beneficial in determining how well the vector administration was restricted to where the injection was made. Additionally, evaluating other tissue and cell types (like the liver or spleen) would possibly identify other sources of expressed ITS01 that could explain the differences in ITS01 concentrations we observed among the individual animals. A previous study had shown evidence of liver transduction in a rhesus macaque following intramuscular administration of an AAV8 vector (24). Lastly, we did not assess the sites of administration at the end of the study, which may have led to insights for the observed ITS01 concentrations that were achieved. Mueller et al. observed an exhausted cytotoxic T cell response to AAV1 capsid at the sites of administration five years after the administration took place (25). Future studies will be needed to determine if this is the case for other or all capsids after i.m. administration.

While not always the target for AAV gene therapies, skeletal muscle tissue provides an excellent system for long-term expression of antibodies. It has been speculated that i.m. administration of AAV vectors could result in years or decades long expression of a transgene. Indeed, Martinez-Navio and Fuchs et al. have demonstrated that a rhesus macaque can express >200 µg/mL of an anti-SIV antibody for nearly seven years (10). That time frame rivals some of the best vaccine-mediated antibody responses to date. Additionally, unlike systemic administration of AAV vectors, AAV vector transduction when delivered by i.m. administration is not as impeded as much by preexisting neutralizing antibodies. Greig et al. showed that AAV8-delivery of an anti-SIV immunoadhesin could express in rhesus macaques with AAV8 neutralizing titers less than 1:360 (18). We routinely pre-screen macaques for preexisting neutralizing antibodies as there is always concern that the presence of preexisting neutralizing antibodies may limit AAV vector transduction. The two macaques that we used in our study that had neutralizing antibodies at a 1:10 titer, rh2813 and r18027, maintained expressed concentrations of ITS01 throughout the study ending with 31 and 151 µg/mL, respectively. However, AAV seroprevalence will most likely need to be considered even for i.m. administration as we presume there to be a limit to what the neutralizing titer can be for efficient vector transduction *via* i.m. administration. In that regard, AAV9 seroprevalence has been described to be lower compared to AAV1 in human populations (26). Overall, i.m. administration remains a viable option for long-term expression of antibodies.

We believe this study can be used as a template for future evaluation of AAV-expressed antibodies from i.m. administration. Johnson et al. first demonstrated that decent expression of anti-SIV immunoadhesins in rhesus macaques using AAV1 vectors (9) but the AAV1.PG9 clinical did not result in any measurable expressed antibody by ELISA (11). This was most likely due to a suboptimal cassette design that utilized two promoters to make the heavy and light chains of PG9. Using the 2A design to make the heavy and light chains of an antibody appears to be the best current solution. Our study first highlights the need to test different 2A peptides to achieve maximal expression. Previous studies examining expression of HIV, influenza, and Ebola antibodies all utilized the F2A peptide (3, 5–8, 12, 14, 15, 27). Our data demonstrate it is possible that better expression could be achieved using a different 2A peptide, like P2A. However, an *in vitro* study suggests that the T2A peptide does improve antibody expression compared to F2A and P2A (21) so it is likely best to test multiple 2A peptides to determine which is best for a specific antibody. Second, our data show that these high concentrations of ITS01 can be achieved from a codon optimized sequence. This suggests that CpG-depleting the transgene cassette may not be necessary when expressing a host-like protein that may not be seen as foreign by the host, a strategy that has been used to decrease host immune responses to AAV vectors (28). Third, spreading out the administration sites likely increases the number of muscle cells that are transduced. Lastly, there is probably a point of diminishing returns for the vector dose used for i.m. administration per site of administration. We used a dose 2.5x10^12^ vg/kg, similar to other macaque studies (18). This was primarily based on the finding from Welles et al. showing no significant difference in antibody expression when injecting 2x10^12^ vg and 2x10^13^ vg in a single i.m. administration site (15). We may need to adjust our thinking for i.m. administration to not only account for vg/kg dose but also vg/dose/site of administration. In a recent clinical trial using AAV8 vectors to deliver the HIV bNAb VRC07, which did utilize the F2A peptide approach, four participants were given 2.5×10^12^ vg/kg of the vector in seven to nine administration sites (16). The authors observed maximum concentrations of VRC07 ranging from 1-3 g/mL. While the number of sites used in those participants is similar to the eight we used in our study, it does not account for the size differences between humans and macaques and indicates possible saturation of the administration site might hinder the number of muscle cells transduced to express the antibody.

Overall, this study demonstrates that high concentrations of antibody can be achieved *via* AAV vectors after i.m. administration in rhesus macaque. The only other study to see these concentrations for multiple macaques was previously reported by Greig et al. (18). Additionally, these concentrations we achieved of ITS01 are almost certainly protective concentrations based on previous studies. If we can translate these results to the clinic, AAV-delivery of HIV-1 bNAbs could provide an alternative approach while a traditional HIV-1 vaccine is developed.

## Materials and methods

### Ethics statement

The 19 rhesus macaques described in this study were Indian-origin rhesus macaques (*Macaca mulatta*), all between two- and five years of age at the time of AAV administration. Macaques were preselected and grouped based on an initial screen for preexisting AAV neutralizing antibodies prior to the study, thus groups were not randomized before the study. No other exclusion criteria were used. All macaques were housed at the Wisconsin National Primate Research Center in accordance with standards set forth by the American Association for Accreditation of Laboratory Animal Care. This study was performed with the approval of the appropriate Institutional Animal Care and Use Committee (IACUC), protocol G005045 (WNPRC). Macaques were housed in pairs for the duration of the study and released back into colony when the study concluded. The macaques used in this study were cared for with standards set forth by the American Association for Accreditation of Laboratory Animal Care and following the guidelines of the Weatherall Report under a protocol approved by the University of Wisconsin Graduate School Animal Care and Use Committee. Macaque weights at the time of AAV administration ranged from 3.0 to 14.1 kg (**Table S1**).

The animal management program of the University of Wisconsin is accredited by the American Association for the Accreditation of Laboratory Animal Care, and it meets NIH standards as set forth in the Guide for the Care and Use of Laboratory Animals (known as “The Guide”). The procedures to avoid unnecessary discomfort, pain, or injury to animals are those prescribed in The Guide. All animals received a complete physical examination before the start of the study. Animals were housed in pairs within the biocontainment facilities and received a daily health check by both animal care technicians and veterinary professional staff. A comprehensive environmental enrichment and psychological well-being plan was in place for the animals in the study, and it is available for inspection by the United States Animal and Plant Health Inspection Service (APHIS) and to officials of any pertinent organization. No animals were lost during this study and all animals were returned to the colony upon completion of the study.

### Cell lines and plasmids

HEK293T cells were obtained from ATCC and grown in DMEM with 10% fetal bovine serum at 37°C. TZM-bl cells were obtained through the NIH AIDS Reagent Program, Division of AIDS, NIAID, NIH from Dr. John C. Kappes, Dr. Xiaoyun Wu, and Tranzyme (29–34) and grown in DMEM with 10% fetal bovine serum at 37°C. AAV.CAG.fLuc was obtained from Addgene (#83281) and described previously (20). Rep/Cap expression plasmids for AAV1, AAV8, and AAV9 were described previously (35). Rep/Cap expression plasmids for AAV-NP22 and AAV-KP1 were described previously (19, 20). ITS01 transgenes were codon optimized with the F2A, P2A, or T2A peptide and synthesized by GenScript. ITS01 transgenes were cloned into an AAV transfer plasmid containing AAV2 ITRs, a CASI promoter, a shorted WPRE (sequence provided by Dr. Alejandro Balazs), and an SV40 polyadenylation signal sequence.

### Antibody production and purification

Production of recombinant ITS01 was performed as previously described (12). Briefly, HEK293T cells in 175 mm plates were transfected with 60 μg total DNA/plate at 80% confluency with PEIpro transfection reagent (Polyplus). Cells were co-transfected with the AAV ITS01 transfer plasmid and a plasmid encoding furin at a 4:1 ratio. At 16 hrs post-transfection, 10% FBS-DMEM media was replaced with serum-free 293 Freestyle media (Invitrogen). Media was collected after 48 hrs, debris was cleared by centrifugation for 10 min at 1,500 g and filtered using 0.45 μm filter flasks (Millipore Sigma). Proteins were isolated with HiTrap columns (Cytiva) and eluted with IgG Elution Buffer (Thermo Scientific) into 1M Tris-HCl Buffer, pH 9.0 (G Biosciences, St. Louis, MO). Buffer was exchanged with PBS and protein concentrated to 1-2 mg/mL with Amicon Ultra Centrifugation Filters (Millipore Sigma). Heavy and light chains of the antibodies were assessed by Coomassie-stained SDS-PAGE. Antibodies were stored at 4°C before use and frozen at -80C for long term storage.

### ITS01 gp120 ELISA

Half-area 96-well Costar Assay Plates (Corning) were coated with 5 μg/mL SIVsmE660 gp120 (Immune Tech New York, NY). Plates were washed with PBS-T (PBS + 0.05% Tween-20) twice and blocked with 2% BSA in PBS for 1 hour at 37°C. Sera samples were unblinded for gp120 ELISA. Sera samples were serially diluted in 2% BSA in PBS and were added to the plate in duplicate. Standard curves were generated by diluting 4 μg/mL purified, recombinant ITS01 protein in 2% BSA then serially diluted and added to the plate in duplicate. Plates were incubated for 1 hour at 37°C. Samples were washed five times with PBS-T and a horseradish peroxidase secondary antibody targeting the IgG Fc (Jackson Immuno Research) was added. Plates were incubated for 1 hour at 37°C and then washed ten times with PBS-T. TMB solution (Fisher) was added and plates were incubated at room temperature until their standard curves developed (typically 5-10 minutes). TMB Stop Solution (Southern Biotech) was then added and absorbance was read at 450 nm by a Perkin Elmer Victor Nivo plate reader (Perkin Elmer). Standard curves were analyzed using GraphPad Prism 6.0 software and used to determine protein titers from sera samples.

### Anti-drug antibody ELISA

Sera samples were unblinded for ADA ELISAs. Sera samples from macaques expressing bNAbs were analyzed against purified, recombinant ITS01 protein. 96 half-well plates were coated with 5 µg/mL of ITS01 in PBS overnight at 4°C. Sera samples were diluted 10-fold and blocked in 2% BSA in PBS for 30 minutes at room temperature. Diluted sera samples were added to the plate and incubated for 1 hour at 37°C. Plates were then washed five times with PBS-T. Anti-transgene antibodies from macaques expressing ITS01 were measured using a secondary antibody detecting the kappa light chain (Millipore) at a 1:8000 dilution. The secondary antibody was added to the plates and plates were incubated for 1 hour at 37°C. Plates were then washed 10 times with PBS-T and TMB solution was added for 10 minutes at room temperature. Stop solution was then added and absorbance at 450 nm was measured as described above.

### AAV neutralization assay

Assays were performed as previously described (35, 36). Serum samples were diluted 1:5 in growth medium and 50 µL was added to a 96-well plate in duplicate. AAV.CAG.fLuc vector was diluted in growth medium to a concentration that would yield >10,000 RLUs (typically 5×10^9^ to 2×10^11^ vg/mL depending on the capsid used) and 50 µL was mixed with 50 µL medium only or each diluted serum sample for a final serum dilution of 1:10. Plates were incubated for 1 hour at 37°C. 100 µL of HEK293T cells were added to each well at a concentration of 30,000 cells/well. Plates were incubated for 24 hours at 37°C. Luciferase was quantified using the britelite plus Reporter Gene Assay system following the manufacturer’s instructions and plates were read on a Perkin Elmer Victor Nivo plate reader.

### AAV vector production and purification for mouse study

AAV9-ITS01 vectors were produced by triple transfection in HEK293T cells. HEK293T cells were split the day before transfection to reach a 60-80% confluency when transfected. Expression plasmids encoding Rep2/Cap9, pHelper, and pAAV.CASI.ITS01 were mixed in serum-free OptiMEM at a 1:1:1 ratio at a total of 60 µg DNA per flask being transfected. Transfections were conducted using PEIpro following the manufacturer’s instructions (Polyplus Transfection). At 3 days post transfection, cells were harvested by adding 0.5M EDTA, pH 8.0 to each flask. Cells and medium were centrifuged at 2000×g for 10 minutes. Cells were washed twice with PBS and then lysed using an AAV lysis buffer (150mM NaCl, 2mM MgCl2, 50mM Tris-HCl pH 8.0). Cells in lysis buffer were subjected to three freeze/thaw cycles. Benzonase (50U/mL) and Triton X-100 (0.01%) were added to the lysate mixture and the mixture was incubated for 1 hour at 37°C. The lysate mixture was centrifuged at 7000×g for 1 hour, the supernatant was collected and filter sterilized with a 0.45 µM filter. POROS Capture Select AAV9 columns were used to purify AAV9 vectors. Eluted vectors were buffer exchanged into sterile PBS and concentrated using Amicon Ultra Centricon Tubes. Vectors were quantified by qPCR with a Roche Lightcylcer 480ii in parallel.

### AAV vector production and purification for NHP study

Production of recombinant AAV at the University of Massachusetts Medical School Vector Core has been previously described (37). Briefly, HEK293T cells were transfected with the AAV transfer plasmid encoding ITS01, a plasmid encoding AAV2 rep and one of the AAV capsids used in this study, and helper plasmid encoding adenovirus genes. After harvesting lysates of transfected cells, AAV was purified through three sequential CsCl centrifugation steps. The vector genome copy number (vg/ml) was determined by qPCR in parallel, except for the second batches of AAV-NP22 and AAV-KP, which were quantified in parallel after separate production. AAV particles were quality controlled by electron microscopy (EM) and the purity of the AAV preparations was verified by silver-stained SDS-PAGE.

### Negative-stain transmission electron microscopy for full capsid analysis

The AAV vectors used for the NHP study were prepared for EM using the conventional negative staining procedure. Formvar-carbon-coated copper grids were treated by glow discharge using a PELCO easiGlow glow discharge cleaning system for 30 seconds at 20 mA. The AAV vectors were negatively stained with 1% (w/v) aqueous uranyl acetate. All images were taken with an FEI Tecnai spirit 12 (FEI Company, Hillsboro, OR), operated at 120 kV. Micrographs were recorded with a Gatan Rio 9 CMOS camera. The capsids in images were counted full and empty using IMAGEJ program (NIH, Bethesda, MD).

### AAV vector administration (mice)

20 male NOD.*Cg-Prkdcscid IL2rgtm1Wjl*/SzJ mice (NSG, strain number 005557) were obtained from The Jackson Laboratory. Mice were inoculated with 5×10^10^ total genome copies of recombinant AAV9 vectors encoding one of the ITS01 variants that use F2A (n=4), P2A (n=8), or T2A (n=8) peptide at a 25 μL volume in the left gastrocnemius muscle. No power analysis was conducted to determine group size. Mice were bled weekly for 4 weeks, and plasma samples were obtained by centrifuging blood samples at 11,000 × *g* for 3 min. Plasma was frozen and kept at −80°C until used analysis by gp120 ELISA. No mice were lost during the study and all mice were euthanized at the end of the study.

### AAV vector administration (rhesus macaques)

All macaques were prescreened for AAV neutralizing antibodies as previously described (35) to all five capsids used in this study prior to the start of the study. Macaques were put into groups of three (AAV1, AAV-NP22, AAV-KP1) or five (AAV8 and AAV9). No power analysis was performed to determine group size. Macaques were inoculated with a dose of 2.5×10^12^ AAV vg/kg. Each macaque received eight i.m. injections: two in the lower left and right quadriceps muscle, two in the upper left and right quadriceps muscle, two in the left and right biceps muscle, and two in the left and right deltoids muscle. Blood draws were taken weekly up to week 4 post administration and then bi-weekly up to 30 weeks post administration.

### TZM-bl neutralization assay

TZM-bl neutralization assays were performed as previously described (38, 39). Briefly, SIVsmE660.A8 pseudovirus was produced by transfecting HEK293T cells seed one day prior with an expression plasmid for SIVsmE660.A8 envelope glycoprotein and the NL4.3deltaEnv expression plasmid at a 1:1 ratio using PEIpro according to the manufacturer’s instructions. Medium was replaced with fresh DMEM (10% FBS) after an overnight incubation. Pseudovirus was harvested 48 hours later with the medium filter sterilized with a 0.22 um syringe filter and stored at -80°C until used. Serum samples were unblinded for analysis. Serial dilutions of heat inactivated serum samples were diluted 1:20 in DMEM (10% FBS) and pre-incubated with SIVsmE660.A8 pseudovirus for 1 hour at 37°C. A positive control consisted of heat inactivated serum prior to AAV administration with a known amount of purified ITS01 added for a starting concentration of 20 µg/mL. The same serum sample was used as a negative control. TZM-bl cells were detached by trypsinization, diluted in DMEM (10% FBS) to 100,000 cells/mL and added to the virus/inhibitor mixture. Cells were then incubated for 44 hours at 37°C. Viral entry was analyzed using Britelite Plus (Perkin Elmer) and luciferase was measured using a Synergy Neo 2 plate read (BioTek).

### Statistical analysis

One-way ANOVA with Tukey’s multiple comparison test and correlation analysis were performed using GraphPad Prism v9.2.0. P values <0.05 were considered significant. Pearson r values >0.7 were considered to be highly correlated and between 0.5 and 0.7 to be moderately correlated.

## Data availability statement

The raw data supporting the conclusions of this article will be made available by the authors, without undue reservation.

## Ethics statement

The animal study was reviewed and approved by University of Wisconsin - Madison.

## Author contributions

MD-G performed experiments. JW performed experiments. JX was responsible for production and quality control of the AAV vectors. KP provided critical reagents and provided input into the study design. EA, KW, JF, and ER were responsible for performing the required procedures and sample collection of the non-human primate study. MK provided critical reagents and provided input into the study design. GG was responsible for production of the AAV vectors. MF conceived the study, acquired funding, and performed the data analysis. MG conceived the study, performed experiments, performed the data analysis, and wrote the initial draft of the manuscript which was reviewed and approved by all coauthors. All authors contributed to the article and approved the submitted version.

## Supplementary material

Supplementary Figure 1 Quality control analysis of AAV vectors used in capsid study. **(A)** Representative negative-stained transmission electron micrograph images for each of the AAV vectors used in capsid study. Note two batches of AAV-NP22 and AAV-KP1 were needed to meet the dose requirements for those groups of macaques. **(B)** Full-empty ratio analysis of the AAV vectors from **(A)**. Images were assessed for full, empty/broken capsids by IMAGEJ software and reported as a percentage of full capsids.

Supplementary Figure 2 Correlation analysis of ITS01 concentrations and AAV vector injection volumes. Correlation plots for AUC of ITS01 concentrations and AAV vector injection volumes for each rhesus macaque are plotted. Statistical analysis for correlation shows the data have no correlation. Pearson r values, P value (two-tailed), and R^2^ values are included.

Supplementary Figure 3 ITS01 concentrations and ADA for each rhesus macaque during the course of the capsid study. ITS01 concentrations (black) and ADA (red) are graphed for each individual rhesus macaque involved in the capsid study. Capsid groups are labeled for each row. Data are the same that was used in **Figures 4**, **5**.
