# Supplementary material for: A strategy for high antibody expression with low anti-drug antibodies using AAV9 vectors
Source: Front Immunol. 2023 Apr 21;14:1105617. doi: 10.3389/fimmu.2023.1105617 (PMC10161250; doi:10.3389/fimmu.2023.1105617)
Supplement: Supplementary file 1 [file Table_1.pdf]

Table S1. Weight and volume of AAV vector injections for each rhesus macaque in the capsid study.

Capsid Group	Animal ID	Weight (kg)	Vector Diluted with PBS	Injection Volume (mL)
AAV1	r16039	6.06	Yes	0.70
	r16042	6.02	Yes	0.70
	r18032	3.03	Yes	0.40
AAV8	r18041	2.8	Yes	0.20
	r18061	3.22	Yes	0.21
	rh2803	5.14	Yes	0.35
	rh2813	11.75	Yes	0.77
	rh2817	11.7	Yes	0.80
AAV9	r18035	3.4	Yes	0.30
	r18051	2.83	Yes	0.13
	rh2773	5.54	Yes	0.50
	rh2793	9.97	Yes	0.44
	rh2819	10.66	Yes	1.00
AAV9-NP22	r18027	3.02	No	0.17
	rh2788	5.55	No	0.32
	rh2815	12.05	No	0.68
AAV-KP1	rh2774	6.77	No	0.27
	rh2781	14.09	No	0.56
	rh2794	6.5	No	0.26
