# Supplementary figures and images for: A strategy for high antibody expression with low anti-drug antibodies using AAV9 vectors
Source: Front Immunol. 2023 Apr 21;14:1105617. doi: 10.3389/fimmu.2023.1105617 (PMC10161250; doi:10.3389/fimmu.2023.1105617)

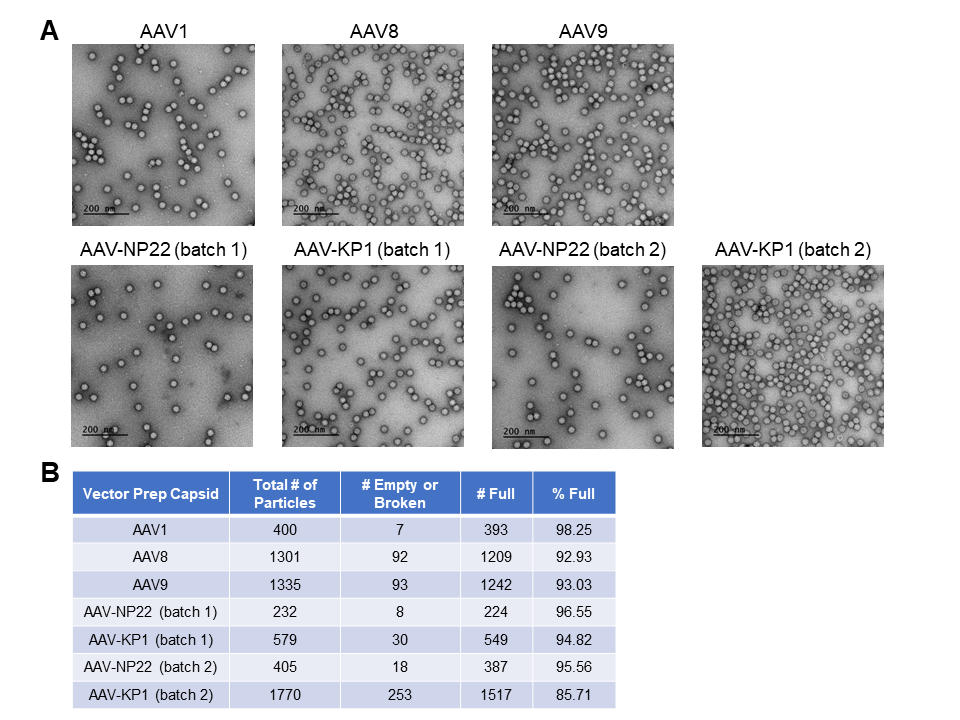

Supplement: Supplementary Figure 1 — Quality control analysis of AAV vectors used in capsid study. (A) Representative negative-stained transmission electron micrograph images for each of the AAV vectors used in capsid study. Note two batches of AAV-NP22 and AAV-KP1 were needed to meet the dose requirements for those groups of macaques. (B) Full-empty ratio analysis of the AAV vectors from (A). Images were assessed for full, empty/broken capsids by IMAGEJ software and reported as a percentage of full capsids. [file Image_1.tif]

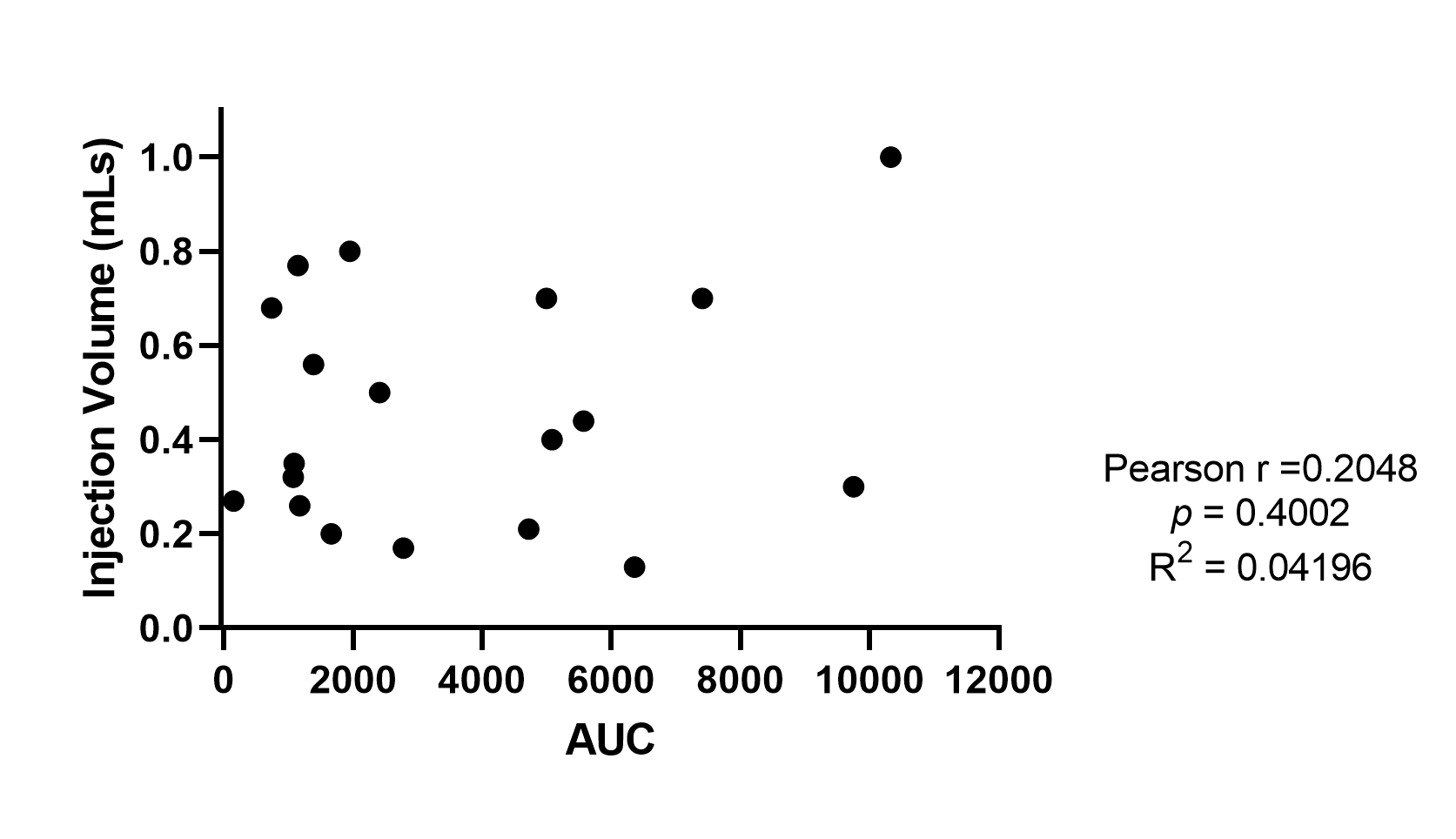

Supplement: Supplementary Figure 2 — Correlation analysis of ITS01 concentrations and AAV vector injection volumes. Correlation plots for AUC of ITS01 concentrations and AAV vector injection volumes for each rhesus macaque are plotted. Statistical analysis for correlation shows the data have no correlation. Pearson r values, P value (two-tailed), and R2 values are included. [file Image_2.tif]

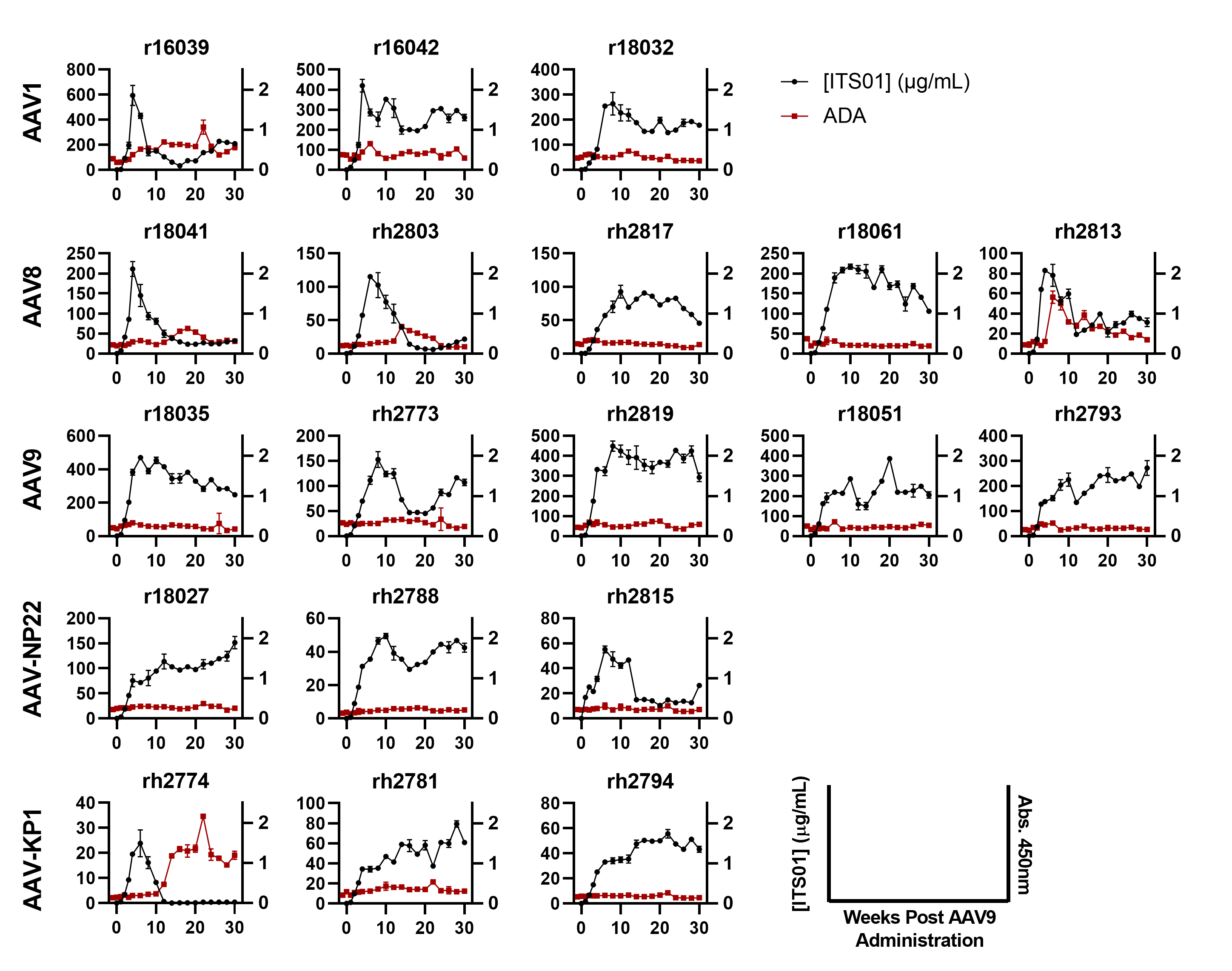

Supplement: Supplementary Figure 3 — ITS01 concentrations and ADA for each rhesus macaque during the course of the capsid study. ITS01 concentrations (black) and ADA (red) are graphed for each individual rhesus macaque involved in the capsid study. Capsid groups are labeled for each row. Data are the same that was used in Figures 4 , 5 . [file Image_3.tif]
